# A Validation Study of Freezing of Gait (FoG) Detection and Machine-Learning-Based FoG Prediction Using Estimated Gait Characteristics with a Wearable Accelerometer

**DOI:** 10.3390/s18103287

**Published:** 2018-09-30

**Authors:** Satyabrata Aich, Pyari Mohan Pradhan, Jinse Park, Nitin Sethi, Vemula Sai Sri Vathsa, Hee-Cheol Kim

**Affiliations:** 1 Department of Computer Engineering/Institute of Digital Anti-Aging Healthcare, Inje University, Gimhae 50834, Korea; satyabrataaich@gmail.com; 2 Department of Electronics and Communication Engineering, IIT Roorkee, Uttarakhand 247667, India; pmpradhan.fec@iitr.ac.in (P.M.P.); nitinsethi.iitr@gmail.com (N.S.); srivathsaeric@gmail.com (V.S.S.V.); 3 Department of Neurology, Haeundae Paik Hospital, Inje University, Busan 47392, Korea; jinsepark@gmail.com

**Keywords:** machine learning, freezing of gait, feature extraction, prediction, wearable accelerometer, gait parameters, mean error rate

## Abstract

One of the most common symptoms observed among most of the Parkinson’s disease patients that affects movement pattern and is also related to the risk of fall, is usually termed as “freezing of gait (FoG)”. To allow systematic assessment of FoG, objective quantification of gait parameters and automatic detection of FoG are needed. This will help in personalizing the treatment. In this paper, the objectives of the study are (1) quantification of gait parameters in an objective manner by using the data collected from wearable accelerometers; (2) comparison of five estimated gait parameters from the proposed algorithm with their counterparts obtained from the 3D motion capture system in terms of mean error rate and Pearson’s correlation coefficient (PCC); (3) automatic discrimination of FoG patients from no FoG patients using machine learning techniques. It was found that the five gait parameters have a high level of agreement with PCC ranging from 0.961 to 0.984. The mean error rate between the estimated gait parameters from accelerometer-based approach and 3D motion capture system was found to be less than 10%. The performances of the classifiers are compared on the basis of accuracy. The best result was accomplished with the SVM classifier with an accuracy of approximately 88%. The proposed approach shows enough evidence that makes it applicable in a real-life scenario where the wearable accelerometer-based system would be recommended to assess and monitor the FoG.

## 1. Introduction

FoG is one of the symptoms that completely immobilize the gait normally seen in the advanced stage of Parkinson’s disease (PD) [1,2]. The best way of defining FoG is the fall that happened because of the improper balance of the posture. It occurs when the PD patient’s feet is “glued to the floor” [3,4]. So far the episodic activity of FoG is assessed using clinical assessment method. Clinicians use two methods for evaluating the FoG. In the first method, the FoG is assessed using specific gait tests such as Unified Parkinson’s disease rating scale (UPDRS) part III or timed-up and go (TUG) test [5,6,7]. UPDRS scale is mostly used to check the severity of the disease. This scale is very broad and primarily divided into six sections. Each section is scored by using a five-point Likert scale. Based on the score of each section, the severity of the disease is assessed [8,9,10,11]. TUG is performed to test the functional performance [12,13]. In this test, the patients have to finish the physical tasks such as rise up from a seated position from a chair, walk 3 m, turn, walk back, and sit down. All these physical tasks have to be finished in a few minutes [14,15]. The second assessment method of FoG is usually carried out by using specific FoG questionnaire, which is collected during the activities of daily living (ADL). Based on the answers responded by the patients and caretakers, the severity and frequency of FoG episode are assessed [16,17,18]. The drawbacks of these methods are mostly observed when these methods are replicated in the real-life environment compared with the controlled laboratory settings. The FoG frequency reduces in a controlled environment compared with the real-life environment [19]. Therefore, researchers have made attempts to find a suitable method to assess and monitor the FoG objectively instead of the subjective method that uses clinical assessment tests. So far the validation study using estimated gait characteristics with wearable accelerometer has not been carried out for detection of FoG. The previous work in literature have not included the gait characteristics as the features for classifying FoG and no FoG patients. This is the first attempt towards validating the gait parameters obtained using the wearable accelerometer with their counterparts from 3D motion capture system. This study also includes FoG prediction based on certain pattern between FoG patients and no FoG patients using machine learning techniques. The features extracted from the signal as well as from the estimated gait characteristics are used for classification. This study also uses a relatively large number of participants with PD. Movement disorder society recommended at least 30 participants for clinical development and testing technique for PD (phase B) [20].

The paper is organized as follows: Section 2 presents the related work. Section 3 describes the materials and methods. Section 4 describes the results. Section 5 discusses the results. Section 6 provides the conclusions.

## 2. Related Work

### 2.1. Digital Mediums to Detect PD

This section reports the previous studies that describe the digital mediums used for measuring the PD. Lipsmeier et al. have proposed a method of using a smartphone to collect the accelerometer and gyroscope data for detecting abnormalities in PD patients during a clinical treatment monitoring. In this study, six activities are monitored on a daily basis, in which the participants have to carry the smartphone in their trouser pocket. They found that the smartphone-based, digital-biomarkers-based clinical treatment is feasible and reliable, and can be recommended for long-term monitoring of the PD patients and for other biomedical research activities [21]. San Luciano et al. have studied PD patients who are asked to draw a spiral on a rectangular space using a digital pen and digitized graphics tablet. The digital spiral drawing is then correlated with the healthy control group to detect the abnormalities in the PD patients [22]. Walker et al. have studied the PD patients and older adults who are asked to perform some drawing task and writing tasks using a digital pen. Analyzing these tasks in terms of abnormalities such as tremor, helps in distinguishing PD patients from patients without PD [23]. Kuhner et al. have proposed a technique that used motion capture system to track the performance online for a healthy group of patients, patients with PD without taking the medicine, and patients with PD taking the medicine while doing certain exercises and tasks such as TUG, hand coordination, and functional reach. They verified the result with the UPDRS scale, and found that the online-based approach is more robust than the offline based approach using clinical scales [24]. In last few years, the wearable device based activity detection is getting a lot of attention for differentiating the PD patients from other neurological disorder patients as well as from healthy older adults. The quality of life of the patients suffering from PD would change if the wearable devices are used with proper software [25]. The popularity of wearable device is more because it is easy to wear, light and less expensive and can facilitate long-term monitoring outside the clinical environment [26,27]. Section 2.2 explains in detail the potential of the wearable sensors for the early detection and monitoring of the PD.

### 2.2. Wearables-Based Activity Detection and Validation for the Diagnosis of Parkinson Disease

This section reports the previous validation studies based on the wearable devices. Coste et al. [28] have proposed a method to detect FoG using inertial sensors placed on the lower limb. The FOG criterion is calculated based on two parameters, stride length and cadence. These two parameters are continuously evaluated using threshold limit. Rodriguez-Martin et al. [29] have proposed a technique to detect the FoG in a home environment based on the features extracted from the data collected from the triaxial accelerometer placed at the waist. The authors compared the performance of the proposed support vector machine (SVM) technique and most comprehensive FoG detection technique (referred as MBFA in that paper) based on two models, generic model and personalized model. The generic model uses the leave-one-patient-out methodology whereas the personalized model uses a modified version of leave-one-patient-out methodology. They have found that personalized FoG model outplays the generic FoG model by 10%. Mileti et al. [30] have proposed a technique that can monitor the gait quality in PD by using wearable sensors. They have used four gait partitioning methods. Out of these partitioning methods, two are based on machine learning techniques, and rest two are based on the thresholds. They have performed these experiments on healthy subjects as well as PD patients with ON and OFF conditions. The results obtained from these gait partitioning methods have a strong correlation with the clinical scale items. Schlachetzki et al. [31] have proposed a system based on the wearable sensors that can quantify gait parameters in an objective manner without the need of specialized clinical environment. They have used this method to distinguish PD group from the control group, based on the gait characteristics such as short step and shuffling of gait. They have validated this method with the clinical scales. Jeon et al. [32] have proposed a method that can measure the tremor severity in PD by using a wearable device that consists of an accelerometer and gyroscope. They have extracted features from the accelerometer and gyroscope signals, and used these features for automatic classification of patients with tremor severity. They have compared their method with the similar state-of-art-based studies, and find their method is effective for the diagnosis of PD. 

The above-mentioned literature confirms the success of various digital mediums for the effective diagnosis of PD. The findings from the previous work helped in designing the proposed approach. Especially the feasibility of wearable device based study for the objective assessment of various characteristics of PD as well as the effectiveness of machine learning techniques for automatic classification of PD characteristics inspired us to design the FoG detection system. In this study, a FoG detection system has been designed, investigated, and validated that can be used in the clinical environment as well as the home environment. 

## 3. Materials and Methods

### 3.1. Data Collection and Experimental Procedure

**Figure 1:**
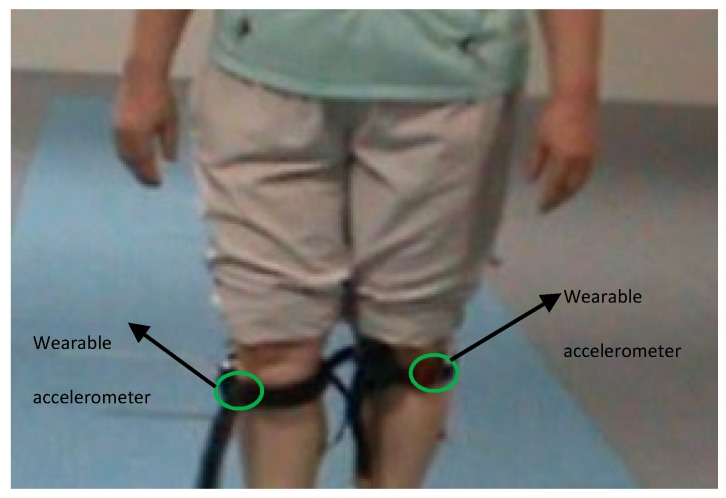
The position of the wearable accelerometer for the clinical experiment.

Fifty-one patients were enrolled in this study. Out of 51 patients, 36 patients have shown symptoms of FoG and rest have not shown any symptom of FoG, although they are diagnosed with PD. All participants wore accelerometers on both knees. Two wearable triaxial accelerometers with sampling frequency of 32 Hz (Fit Meter, Fit.Life, Suwon, Korea) were used. The triaxial accelerometer measures body movements in all directions: anterior–posterior, medio-lateral, and vertical. It is small and lightweight (35 mm × 35 mm × 13 mm and 13.7 gm). It is sensitive to acceleration from −8 g to 8 g, allowing for monitoring of almost all human physical activities. The wearable accelerometers are placed at the left as well as right knee, and tightly fastened using straps. The position of accelerometer at the left knee, as well as the right knee, is 34 cm above the ground as shown in Figure 1. The sensors were tightly fastened to both the knees using straps, and therefore the probability of falling down is negligible. Two assistant nurses were accompanied with the participants during all test procedures for recording, monitoring and preventing the fall of accelerometer sensor. After examination, the neurologist analyzed the video clip, and monitored the state of the sensors.

All participants gave their consent to participate in this study. This study was approved by the institutional review board at Haeundae Paik Hospital, Busan, South Korea (IRB No. 2017-01-028). The criteria for inclusion are as follows:

(1) Patients who were diagnosed with PD clinically [33],
(2) Age between 40 and 80,
(3) Hoehn and Yahr stages 2–4,
(4) Patients who showed gait disturbance.

Demographic data of the patients are as follows: Mean age is 71.60 ± 9.01. Mean disease duration is 34.89 ± 27.77 and mean UPDRS motor scale is 21.02 ± 11.21. Mean H&Y stage is a relatively early stage (2.08 ± 0.76). All participants walked in a room where the motion was captured using a three-dimensional (3D) motion capture system (VICON, Oxford, UK) environment. The walking test in this study was carried out in a rectangular area measuring 8 m × 6 m. The motion was captured using 12 infrared cameras available in the 3D motion capture system (VICON). All participants were asked to walk along the 6 m track for three times. The quantified spatiotemporal parameters include step time, stride time, step length, stride length, and walking speed.

### 3.2. Study Design

This study is a prospective, cross-sectional study. In this study, the collected data has been processed using a computer with following specification: window 10, 3.60 GHz 64-Bit Intel Core i7-7700 processor and 8 GB RAM. Rawassizadeh et al. [34] have proposed on-device analysis for mobile and wearable devices without using the cloud, which has the benefits in terms of energy efficiency, response time, reliability, etc. over the cloud based processing. In the proposed work, the benefits of cloud based processing are not substantial looking at the computation time (approx. 2.3 s), cost and complexity. Although the proposed study uses a computer for processing the data, this task could also be performed using the on-device processor [34]. In this study, the time delay has been optimized based on the method proposed by Chang et al. [35] in which frequency domain information, as well as time domain information, is used for determining the freezing index.

The gait parameters including step time, stride time, step length, stride length, and walking speed were calculated using the data obtained from a triaxial wearable accelerometer. Previous researchers have reported the importance of the five parameters for the detection of PD. Hollman et al. [36] have proposed five major domains of gait based on factor analysis. (1) Rhythm domain including step and stride time; (2) phase domain characterized by temporophasic domain of gait cycle; (3) variability domain including step variability; (4) pace domain including gait speed, stride and step length; (5) base of support domain characterized by step width. Coste et al. [28] have discussed the importance of stride length in detecting the changes during FoG. Alcock et al. [37] have proposed that the increase in step length prevents the risk of fall in PD as well as in case of old adults. Schlachetzki et al. [31] have discussed the importance of spatiotemporal gait parameters such as stride length, stride time and gait velocity in distinguishing the PD patients from healthy older adults. Del et al. [38] have emphasized on the importance of variability and asymmetry in step time, step length, and step velocity in distinguishing the PD patients from healthy older groups. The literature survey shows that the aforementioned five gait parameters have been widely used in the gait analysis, and hence also considered in the proposed study. 

The percentage rate of error between gait parameters obtained from accelerometer data and those obtained from 3D motion capture system is calculated as follows:

$$\begin{array}{l} \mathrm{Mean}\;\mathrm{error}\;\mathrm{rate}\left(\%\right)\\ =\frac{\left(Gait\;parameters\;from\;accelerometer\right)-\left(Gait\;parameter\;from\;3D\;motion\;capture\right)}{\left(Gait\;parameter\;from\;3D\;motion\;capture\right)}*100\;\left(\%\right) \end{array}$$

The primary endpoint is to estimate the mean error rate for gait parameters obtained from wearable accelerometer compared to those obtained from 3D motion capture system, and to evaluate the correlation between step time, stride time, step length, stride length, and walking speed estimated from both the methods. The secondary endpoint is to compare the performance of the classifiers in predicting FoG and no FoG for the same dataset.

The Minitab version 18 is used for statistical analysis. Pearson’s correlation coefficients between gait parameters obtained from wearable accelerometer data, various clinical scales, and 3D motion capture system were used for concurrent validity. MATLAB (version R2018a) was used for development of validation and classification algorithm.

### 3.3. Gait Cycle Detection with Wearable Accelerometer

The gait cycle is detected using the method proposed by Del et al. [39]. The first step is to preprocess the raw data to eliminate the offsets and misalignments created due to the orientation of the wearable device. The triaxial (X, Y, Z) to horizontal-vertical transformation approach [40] has been used to correct these errors. The next step is to filter the preprocessed data using a low pass filter. A fourth-order Butterworth Filter with a cut off frequency of 15 Hz is used. The filtered acceleration signal is smoothed by integration. The heel strike events in a gait cycle, which also represent the initial contacts (ICs) of the leg, can be detected from the minima of the smoothed acceleration signal. To achieve this, the first order derivative of the smoothed acceleration signal is calculated using the Gaussian continuous wavelet transform. The points of minima in the resulting signal represent the ICs of the leg. 

### 3.4. Estimation of Spatiotemporal Gait Parameters and Feature Selection

**Figure 2:**
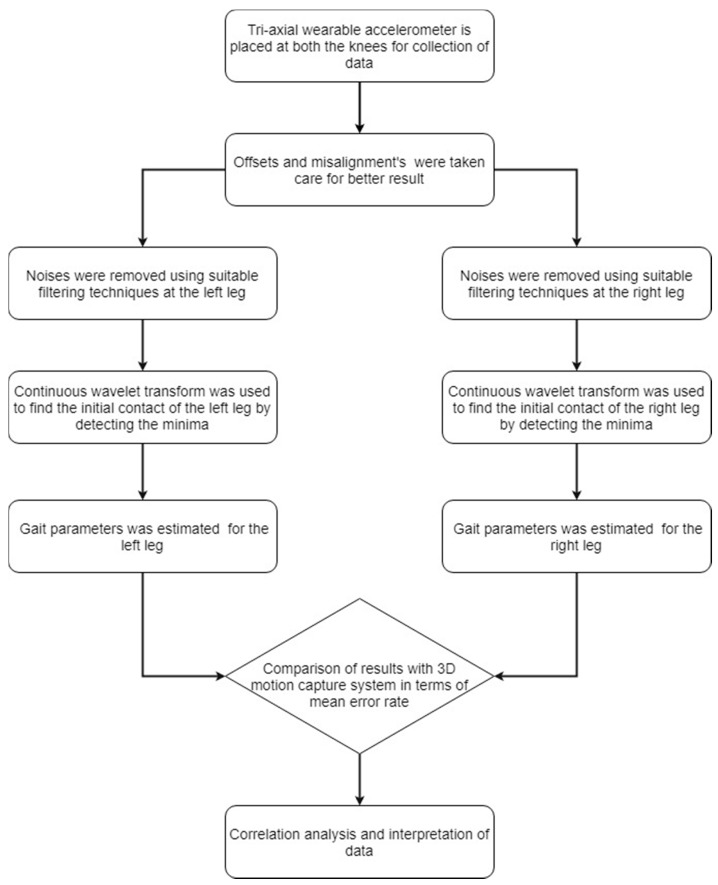
Flowchart for wearable based validation in FoG patients.

The flowchart for proposed accelerometer-based validation of gait parameters in FoG patients is shown in Figure 2. Five gait parameters are calculated from accelerometer data, which include step time, stride time, step length, stride length and walking speed. The *IC* events within the gait cycle are used to estimate the five spatiotemporal parameters. The equations used to estimate the step time and stride time are as follows:

Step Time(i)=IC(i+1)−IC(i)

**Figure 3:**
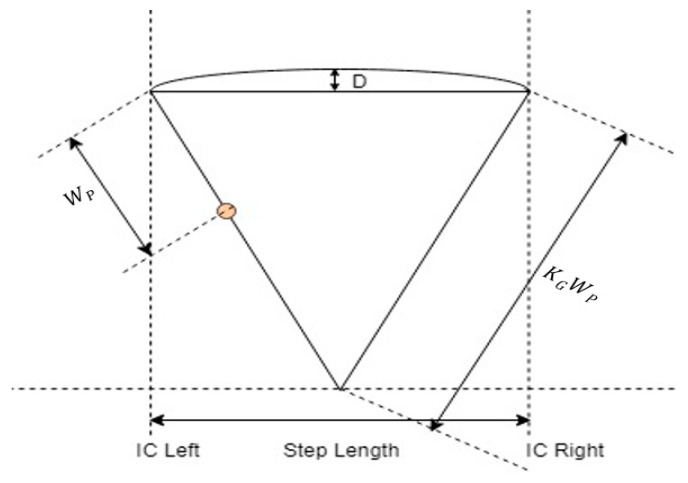
Extension of an inverted pendulum model for calculation of step length.

Stride Time(i)=IC(i+2)−IC(i)

where *i* represents the index of the sequence of *IC* events in the signal. The inverted pendulum model [31] is extended for calculation of step length as shown in Figure 3.

The equations used to estimate the step length and stride length are as follows:

$$Step\;Length=K_{G}*2\sqrt{\left(2W_{P}D-D^{2}\right)}$$

Stride Length=2∗Step Length

where $W_{p}$ is the height of the wearable accelerometer from the ground, $K_{G}$ is a multiplying factor to map position of the wearable accelerometer to that of center of mass in inverted pendulum model, and *D* represents the change in the height of the wearable accelerometer that can be computed using double integration of the vertical acceleration between two consecutive *IC* events. In order to avoid iterative time-consuming training time required for finding individual multiplying factor for each patient, a generic multiplying factor $K_{G}$ = 4 is used in this study. The equation used to estimate the walking speed is as follows:

$$Walking\;Speed=\frac{mean\;step\;length}{mean\;step\;time}$$

In addition to the aforementioned five spatiotemporal gait parameters, nine conventional statistical features are also used in this study for FOG classification. The 14 features along with their description are listed in Table 1. Principal Component Analysis (PCA) of the original data, consisting of 14 features, resulted in 14 principal components. The principal components are described in terms of total variance in the original data, which should be at least 95% [41,42]. In the proposed study, all 14 principal components provide a variance of more than 95%, and hence all 14 principal components were selected as features. Therefore, in total, this study deploys a 28-dimensional feature vector for FOG classification.

### 3.5. Need of Machine Learning Techniques and Its Perfromance Measurement

In recent years with the challenge of making a real-time system, machine learning techniques have a huge role to play in terms of detection, monitoring, and prediction from large number of data. Rawassizadeh et al. [43] have used machine-learning techniques to analyze the impact of semantic abstraction on data prediction. Rehman et al. [44] have discussed data mining or machine-learning techniques for extracting important information from the personalized devices such as mobile phone and wearable devices. In the proposed work, machine learning techniques are used to analyze the impact of extracted gait features on the predictability of FoG. Supervised machine learning techniques are recommended for the personalized device data such as mobile phone data or wearable device data because the classes for the disease dataset are known in advance. Banaee et al. [45] have shown that supervised learning technique such as support vector machine (SVM), neural network (NN), and decision tree (DT) based classifiers can be successfully used for decision-making tasks in the healthcare domain. Howcroft et al. [46] have found that SVM, Naïve Bayes (NB), NN are the best classification techniques for the classification of fall risk. Casto et al. [47] have discussed the potential of the supervised machine learning techniques such as SVM, NB, DT for the categorization of different human activity while connected to Internet of Things (IoT). Looking at the wide usage of SVM, k-NN, NB, DT in the literature, the proposed study uses these four classifiers for classifying patients with FoG or no FoG. A well-designed FoG detection system needs a fast and effective algorithm that can work as a binary classification system to classify FoG patients from no FoG patients. The performance of a system can be measured by using the following performance metrics such as accuracy, sensitivity, and specificity. Accuracy is measured on the basis of the correct distinction between FoG patients and no FoG patients. Sensitivity is defined by the ability of the system to detect FoG, and expressed as the ratio of true positives to the total number of FoG. Specificity is defined by the ability of the system to detect FoG only when the system detect. True positives (TP) are the samples or data points belonging to FoG, correctly predicted as FoG. False negatives (FN) are the samples or data points belonging to FoG, but predicted as no FoG. True negatives (TN) are the samples or data points belonging to no FoG and correctly predicted as no FoG. False positives (FP) are the samples or data points belonging to no FoG, but wrongly predicted as FoG. In the proposed study, stratified train-validation split has been used in the ratio of 70:30. Therefore, data of 36 out of 51 patients have been used for training, and remaining data of 15 patients have been used for validation. For validation, 10 patients with symptoms of FoG and 5 patients with no symptoms of FoG were considered. The proposed model has been built based on training and validation.

## 4. Results

**Figure 4:**
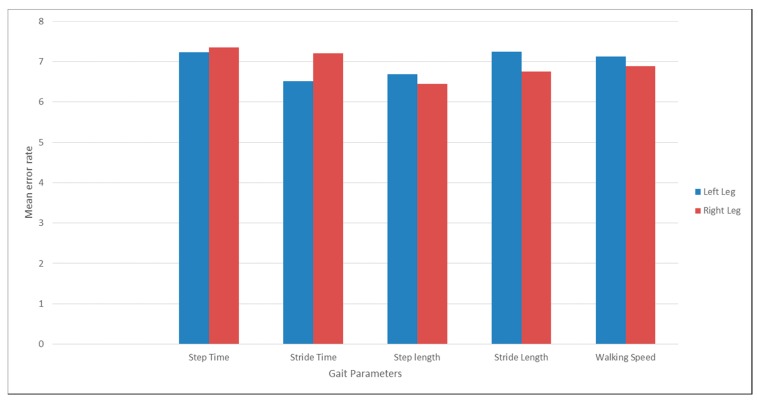
Mean error rate for gait parameters for left and right legs obtained from wearable accelerometer-based approach and 3D motion capture system.

**Figure 5:**
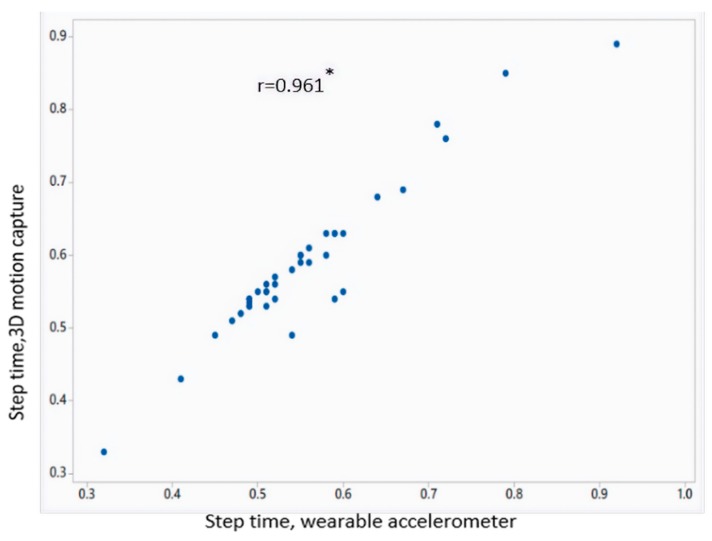
Correlation plot of step time estimated from wearable accelerometer-based approach and 3D motion capture system (* *p* < 0.01).

**Figure 6:**
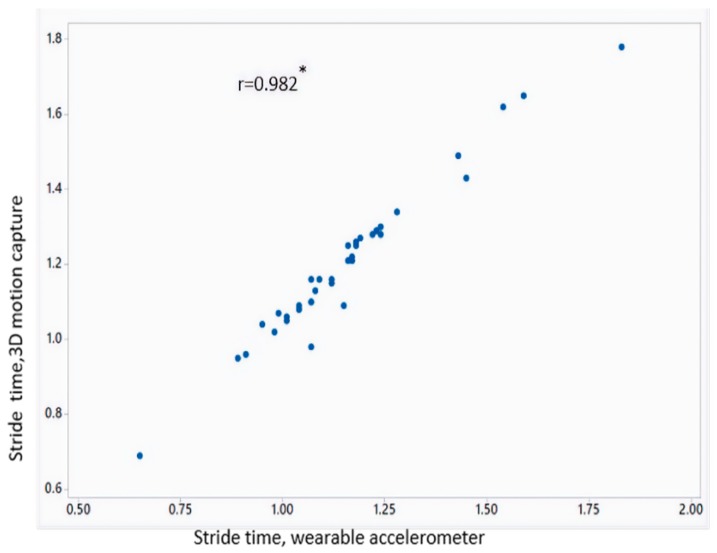
Correlation plot of stride time estimated from wearable accelerometer-based approach and 3D motion capture system (* *p* < 0.01).

**Figure 7:**
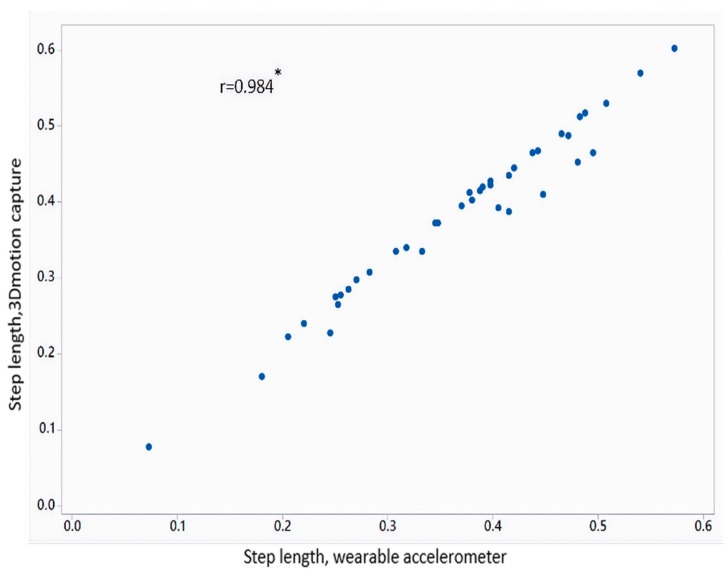
Correlation plot of step length estimated from wearable accelerometer-based approach and 3D motion capture system (* *p* < 0.01).

**Figure 8:**
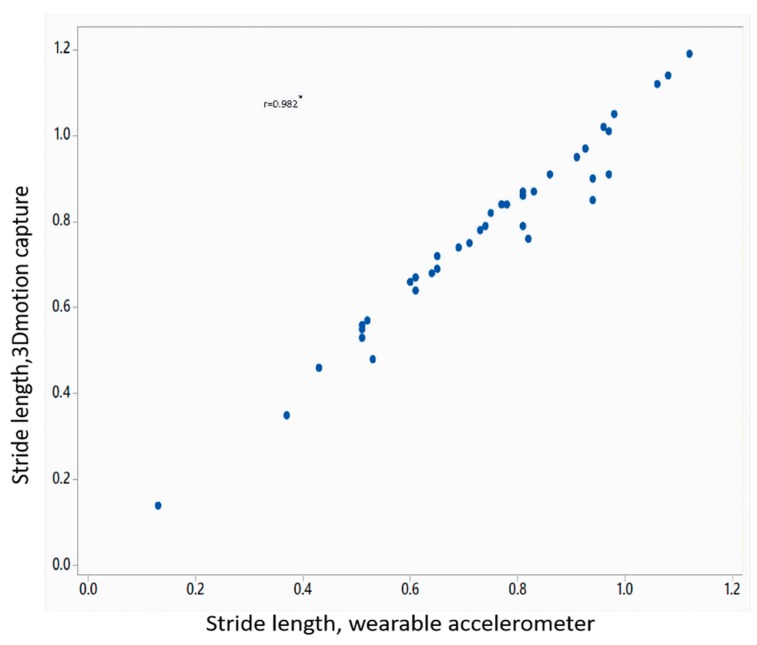
Correlation plot of stride length estimated from wearable accelerometer-based approach and 3D motion capture system (* *p* < 0.01).

**Figure 9:**
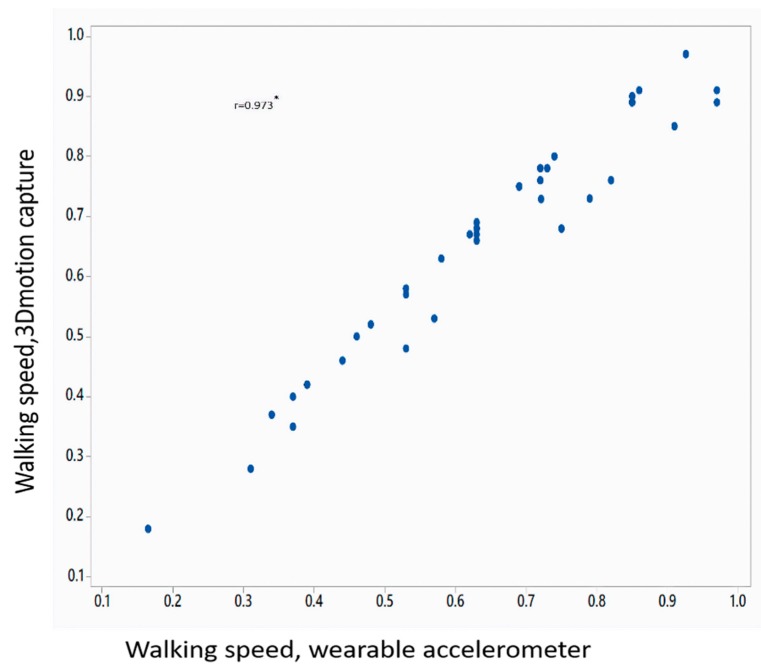
Correlation plot of walking speed estimated from wearable accelerometer-based approach and 3D motion capture system (* *p* < 0.01).

Table 2 shows the mean value of gait parameters estimated from the wearable accelerometer data and 3D motion capture system. Figure 4 shows the bar plot of the mean error rate for gait parameters for left and right legs obtained from wearable accelerometer-based approach and 3D motion capture system. The five gait parameters obtained from the wearable accelerometer-based approach and those obtained from 3D motion capture system (Figure 5, Figure 6, Figure 7, Figure 8 and Figure 9) are highly correlated. Estimated step time, stride time, step length, stride length, and walking speed using the proposed approach showed a good correlation with the counterparts obtained from the 3D motion capture system i.e., (*r* = 0.961, *p* < 0.01), (*r* = 0.982, *p* < 0.01), (*r* = 0.984, *p* < 0.01), (*r* = 0.982, *p* < 0.01), (*r* = 0.973, *p* < 0.01), respectively. The performances of four machine-learning techniques are compared in terms of accuracy, sensitivity, and specificity. The results obtained using the four classifiers are shown in the Table 3. The classification algorithms are implemented in a MATLAB R2018a environment on a computer with following specifications: Windows 10 operating system, 3.6 GHz 64-Bit Intel Core i7-7700 processor, and 8 GB RAM.

**Figure 10:**
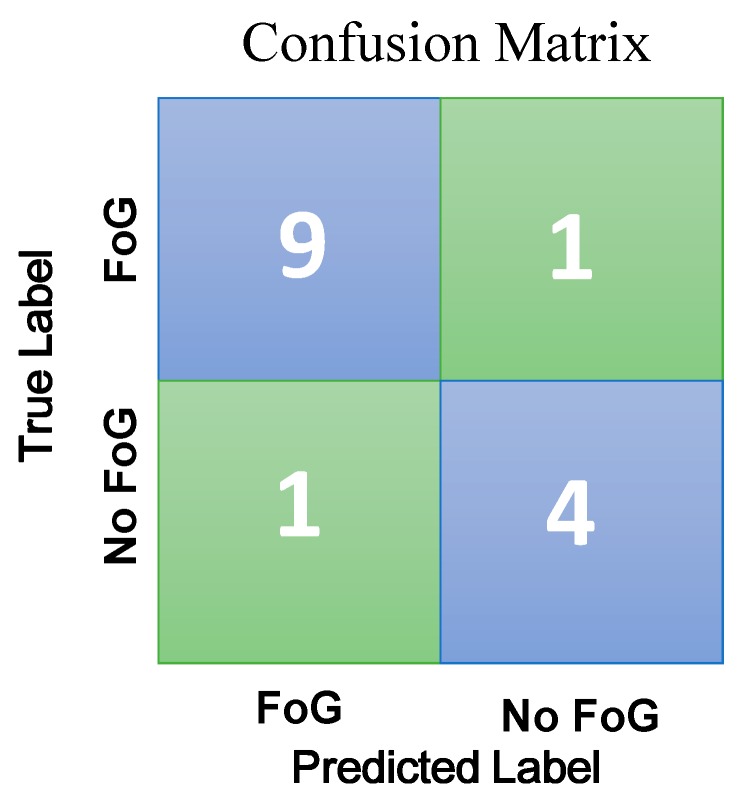
Confusion matrix for the proposed approach.

The mean error rate was found below 10%. The SVM classifier with radial basis function provides the highest accuracy of 91.42%. It indicates that out of 15 patients that includes both the classes we could predict 46 patient correctly. It also gives the highest sensitivity as well as specificity of 90.89% and 91.21% respectively. This result indicates that out of 36 FoG patients, the system correctly identified 32 as FoG and out of 15 no FoG patients, the system correctly identified 14 as no FoG patients. The FoG classification confusion matrix is shown in Figure 10.

## 5. Discussion

This study proposed another novel approach for quantifying the gait characteristics for FoG detection and automatic FoG detection system in PD using machine learning techniques. We have incorporated previous researcher’s findings to improve the result. Coste et al. [28] have measured the gait parameters from video recording, and then used those parameters for detecting the FoG. They have emphasized that the gait parameters such as stride length and cadence have a huge role to play in detection of FoG. In the proposed study, those parameters have been included for FoG detection. Rodriguez-Martin et al. [29] have extracted features from the data generated from the wearable accelerometer, and used them for the detection of FoG. In the proposed work, features have been extracted from the data from a wearable accelerometer for classifying FoG patients from no FoG. Although the wearable sensor-based study has been carried out for the measurement of gait parameters in PD patients, this is the first validation study for FoG detection among advanced stage of PD patients using estimated gait parameters with wearable accelerometer as well as the 3D motion capture system. The previous study [48] that uses wearable sensors and performs validation study, has found the mean error rate of less than 6% for the stride length among the healthy subjects, and 10.3% for the stride length among PD group. The results obtained using a wearable accelerometer-based approach are in accordance with previous reports.

The novelties of the proposed study are as follows: (1) This validation study involves 39 patients which is well above the minimum 30 number of sample size suggested in [20], which implies that the proposed study could be recommended for a real-life scenario. (2) This study uses only the accelerometer data for estimating spatiotemporal gait parameters. The good accuracy of the estimated parameters motivates the exclusion of gyroscopic data for this study. Therefore, this validation study provides a low-cost alternative for assessing gait parameters in the FoG patients. (3) This study demonstrates that gait parameters estimated from accelerometer data and those obtained from 3D motion capture system are highly correlated for concurrent validity. (4) This is the first classification study that includes gait parameters for classification whereas the previous study [29] uses only the axes features for classification.

The wearable accelerometer showed acceptable accuracy in estimating gait parameters in FoG patients. The results show that the mean error rate for gait parameters obtained from accelerometer data is approximately 8% compared to those obtained from 3D motion capture system. Compared to the existing approaches for FoG detection using machine-learning techniques, the classification results obtained using the proposed approach outweighs their performance. Further, the number patients considered in this study is more compared to the previously reported studies. The performance comparison of different FoG detection methods using machine-learning techniques is shown in the Table 4. 

## 6. Conclusions

In this paper, low-cost wearable sensors were used to assess the feasibility of a measurement system before implementing it in the practical environment. An excellent level of agreement was found between the gait parameters obtained from wearable accelerometer-based method, 3D motion capture system and clinical scales, with less than 10% error. The wearable triaxial accelerometer-based method could be considered as a low-cost alternative method for detection of FoG in PD patients. Good classification accuracy was found for the discrimination of FoG and no FoG patients. The good classification accuracy helps in monitoring the FoG and no FoG Patients. The wearable accelerometer can enable long-term monitoring and assessment of gait in a home-based free-living environment. In the future, the validation methods will be tested with bigger sample sizes as well as in real-life scenarios. Further, advanced machine learning techniques will be employed for better classification performance. This kind of study will help clinicians in assessing and monitoring the FoG patients. 

## Figures and Tables

**Table 1:** Computed features and their brief description.

Feature Variable	Feature Name	Description
avg_step_time	Average step time	Average time elapsed for each step
avg_stride_time	Average stride time	Average time elapsed for each stride
avg_step_length	Average step length	Average distance covered in each step
avg_stride_length	Average stride length	Average distance covered in each stride
walking_speed	Walking speed	Gait velocity or speed of walking
sigma_x	Standard deviation of acceleration along *x*-axis	Measure for signal spreading characterized as mean deviation of the signal compared to the average
sigma_y	Standard deviation of acceleration along *y*-axis	
sigma_z	Standard deviation of acceleration along *z*-axis	
S_xy	Zeroth-Lag cross-correlation coefficient between accelerations along *x*-axis and *y*-axis	Agreement or similarity between acceleration signals
S_xz	Zeroth-Lag cross-correlation coefficient between accelerations along *x*-axis and *z*-axis	
S_yz	Zeroth-Lag cross-correlation coefficient between accelerations along *y*-axis and *z*-axis	
harmonic_x	Harmonic ratio for acceleration along *x*-axis	Harmonic composition of the accelerations for a given stride
harmonic_y	Harmonic ratio for acceleration along *y*-axis	
harmonic_z	Harmonic ratio for acceleration along *z*-axis	

**Table 2:** Mean value of gait parameters obtained from wearable accelerometer-based estimation and 3D motion capture system.

S. No.	Parameters	Mean Value (3D Motion Capture)	Mean Value (Algorithm)	Mean Error Rate (%)
1	Step Time (s)	0.53	0.57	7.64 ± 2.41
2	Stride Time (s)	1.17	1.13	5.45 ± 3.57
3	Step length (cm)	0.38	0.35	6.35 ± 2.45
4	Stride Length (cm)	0.73	0.71	6.25 ± 2.81
5	Walking Speed (cm/s)	0.68	0.65	6.52 ± 3.23

**Table 3:** Result of the four classifiers after 10 successive simulations.

**(a) SVM**											
**Simulation**	**1**	**2**	**3**	**4**	**5**	**6**	**7**	**8**	**9**	**10**	**Mean**
Accuracy (%)	85.71	88.57	88.57	91.42	88.57	88.57	91.42	88.57	88.57	91.42	89.139
Sensitivity (%)	85.11	87.91	87.91	90.89	87.91	87.91	90.89	87.91	87.91	90.89	88.524
Specificity (%)	85.34	88.12	88.12	91.21	88.12	88.12	91.21	88.12	88.12	91.21	88.769
**(b) *k*-NN**											
**Simulation**	**1**	**2**	**3**	**4**	**5**	**6**	**7**	**8**	**9**	**10**	**Mean**
Accuracy (%)	85.29	85.29	85.29	82.35	85.29	85.29	82.35	85.29	85.29	85.29	84.702
Sensitivity (%)	84.97	84.98	84.97	82.11	84.98	84.98	82.12	84.98	84.98	84.98	84.405
Specificity (%)	84.98	84.98	84.98	82.23	84.98	84.98	84.98	84.98	84.98	84.98	84.705
**(c) Decision Tree**											
**Simulation**	**1**	**2**	**3**	**4**	**5**	**6**	**7**	**8**	**9**	**10**	**Mean**
Accuracy (%)	85.88	87.12	87.12	85.88	85.88	87.12	87.12	87.12	87.12	87.12	86.748
Sensitivity (%)	85.56	86.98	86.97	85.56	85.56	86.97	86.97	86.97	86.97	86.97	86.548
Specificity (%)	85.67	86.99	86.99	85.67	85.67	86.99	86.99	86.99	86.99	86.99	86.594
**(d) NB**											
**Simulation**	**1**	**2**	**3**	**4**	**5**	**6**	**7**	**8**	**9**	**10**	**Mean**
Accuracy (%)	83.12	82.89	82.89	83.12	83.12	82.89	83.12	83.12	83.12	83.12	83.051
Sensitivity (%)	82.92	82.48	82.47	82.92	82.92	82.48	82.92	82.92	82.92	82.92	82.787
Specificity (%)	82.97	82.54	82.53	82.97	82.97	82.54	82.97	82.97	82.97	82.97	82.84

**Table 4:** Performances of machine-learning-based FoG detection studies.

Author	Number of Patients	Classification Accuracy (%)
Our Work	51	91.42
Handojoseno [49]	10	75
SAAD [41]	10	88

## References

[1] Fahn S. (1995). The freezing phenomenon in parkinsonism. Adv. Neurol..

[2] Giladi N., Treves T.A., Simon E.S., Shabtai H., Orlov Y., Kandinov B., Paleacu D., Korczyn A.D. (2001). Freezing of gait in patients with advanced Parkinson’s disease. J. Neural Transm..

[3] Bloem B.R., Hausdorff J.M., Visser J.E., Giladi N. (2004). Falls and freezing of gait in Parkinson’s disease: A review of two interconnected, episodic phenomena. Mov. Disord..

[4] Moreau C., Defebvre L., Bleuse S., Blatt J.L., Duhamel A., Bloem B.R., Destée A., Krystkowiak P. (2008). Externally provoked freezing of gait in open runways in advanced Parkinson’s disease results from motor and mental collapse. J. Neural Transm..

[5] Podsiadlo D., Richardson S. (1991). The timed “Up & Go”: A test of basic functional mobility for frail elderly persons. J. Am. Geriatr. Soc..

[6] Schaafsma J.D., Balash Y., Gurevich T., Bartels A.L., Hausdorff J.M., Giladi N. (2003). Characterization of freezing of gait subtypes and the response of each to levodopa in Parkinson’s disease. Eur. J. Neurol..

[7] Snijders A.H., Haaxma C.A., Hagen Y.J., Munneke M., Bloem B.R. (2012). Freezer or non-freezer: Clinical assessment of freezing of gait. Park. Relat. Disord..

[8] Fahn S. (1987). Members of the UPDRS development committee. Unified Parkinson’s disease rating scale. Recent Dev. Parkinson’s Dis..

[9] Martínez-Martín P., Gil-Nagel A., Gracia L.M., Gómez J.B., Martinez-Sarries J., Bermejo F., Cooperative Multicentric Group (1994). Unified Parkinson’s disease rating scale characteristics and structure. Mov. Disord..

[10] Baas H., Stecker K., Fischer P.A. (1993). Value and appropriate use of rating scales and apparative measurements in quantification of disability in Parkinson’s disease. J. Neural Transm. Park. Dis. Dement. Sect..

[11] Goetz C.G., Stebbins G.T., Shale H.M., Lang A.E., Chernik D.A., Chmura T.A., Ahlskog J.E., Dorflinger E.E. (1994). Utility of an objective dyskinesia rating scale for Parkinson’s disease: Inter-and intrarater reliability assessment. Mov. Disord..

[12] Steffen T.M., Hacker T.A., Mollinger L. (2002). Age-and gender-related test performance in community-dwelling elderly people: Six-Minute Walk Test, Berg Balance Scale, Timed Up & Go Test, and gait speeds. Phys. Ther..

[13] Lusardi M.M., Pellecchia G.L., Schulman M. (2003). Functional performance in community living older adults. J. Geriatr. Phys. Ther..

[14] Viccaro L.J., Perera S., Studenski S.A. (2011). Is timed up and go better than gait speed in predicting health, function, and falls in older adults?. J. Am. Geriatr. Soc..

[15] Nocera J.R., Stegemöller E.L., Malaty I.A., Okun M.S., Marsiske M., Hass C.J. (2013). Using the Timed Up & Go test in a clinical setting to predict falling in Parkinson’s disease. Arch. Phys. Med. Rehabil..

[16] Giladi N., Shabtai H., Simon E.S., Biran S., Tal J., Korczyn A.D. (2000). Construction of freezing of gait questionnaire for patients with Parkinsonism. Park. Relat. Disord..

[17] Giladi N., Tal J., Azulay T., Rascol O., Brooks D.J., Melamed E., Oertel W., Poewe W.H., Stocchi F., Tolosa E. (2009). Validation of the freezing of gait questionnaire in patients with Parkinson’s disease. Mov. Disord..

[18] Nilsson M.H., Hariz G.M., Wictorin K., Miller M., Forsgren L., Hagell P. (2010). Development and testing of a self administered version of the Freezing of Gait Questionnaire. BMC Neurol..

[19] Nieuwboer A., Weerdt W.D., Dom R., Lesaffre E. (1998). A frequency and correlation analysis of motor deficits in Parkinson patients. Disabil. Rehabil..

[20] Maetzler W., Klucken J., Horne M. (2016). A clinical view on the development of technology-based tools in managing Parkinson’s disease. Mov. Disord..

[21] Lipsmeier F., Taylor K.I., Kilchenmann T., Wolf D., Scotland A., Schjodt-Eriksen J., Cheng W.Y., Fernandez-Garcia I., Siebourg-Polster J., Jin L. (2018). Evaluation of Smartphone-Based Testing to Generate Exploratory Outcome Measures in a Phase 1 Parkinson’s Disease Clinical Trial. Mov. Disord..

[22] San Luciano M., Wang C., Ortega R.A., Yu Q., Boschung S., Soto-Valencia J., Bressman S.B., Lipton R.B., Pullman S., Saunders-Pullman R. (2016). Digitized spiral drawing: A possible biomarker for early Parkinson’s disease. PLoS ONE.

[23] Walker R.W., Zietsma R., Gray W.K. (2014). Could a new sensory pen assist in the early diagnosis of Parkinson’s?. Expert Rev. Med. Devices.

[24] Kuhner A., Schubert T., Maurer C., Burgard W. An online system for tracking the performance of Parkinson’s patients. Proceedings of the 2017 IEEE/RSJ International Conference on Intelligent Robots and Systems (IROS).

[25] Prasad R., Babu S., Siddaiah N., Rao K. (2016). A review on techniques for diagnosing and monitoring patients with Parkinson’s disease. J. Biosens. Bioelectron..

[26] Han J.H., Lee W.J., Ahn T.B., Jeon B.S., Park K.S. Gait analysis for freezing detection in patients with movement disorder using three dimensional acceleration system. Proceedings of the 25th Annual International Conference of the IEEE Engineering in Medicine and Biology Society.

[27] Moore S.T., Yungher D.A., Morris T.R., Dilda V., MacDougall H.G., Shine J.M., Naismith S.L., Lewis S.J. (2013). Autonomous identification of freezing of gait in Parkinson’s disease from lower-body segmental accelerometry. J. Neuroeng. Rehabil..

[28] Azevedo Coste C., Sijobert B., Pissard-Gibollet R., Pasquier M., Espiau B., Geny C. (2014). Detection of freezing of gait in Parkinson disease: Preliminary results. Sensors.

[29] Rodríguez-Martín D., Samà A., Pérez-López C., Català A., Arostegui J.M.M., Cabestany J., Bayés À., Alcaine S., Mestre B., Prats A. (2017). Home detection of freezing of gait using support vector machines through a single waist-worn triaxial accelerometer. PLoS ONE.

[30] Mileti I., Germanotta M., Di Sipio E., Imbimbo I., Pacilli A., Erra C., Petracca M., Rossi S., Del Prete Z., Bentivoglio A.R. (2018). Measuring Gait Quality in Parkinson’s disease through Real-Time Gait Phase Recognition. Sensors.

[31] Schlachetzki J.C., Barth J., Marxreiter F., Gossler J., Kohl Z., Reinfelder S., Gassner H., Aminian K., Eskofier B.M., Winkler J. (2017). Wearable sensors objectively measure gait parameters in Parkinson’s disease. PLoS ONE.

[32] Jeon H., Lee W., Park H., Lee H., Kim S., Kim H., Jeon B., Park K. (2017). Automatic classification of tremor severity in Parkinson’s disease using a wearable device. Sensors.

[33] Hughes A.J., Daniel S.E., Blankson S., Lees A.J. (1993). A clinicopathologic study of 100 cases of Parkinson’s disease. Arch. Neurol..

[34] Rawassizadeh R., Pierson T.J., Peterson R., Kotz D. (2018). NoCloud: Exploring Network Disconnection through On-Device Data Analysis. IEEE Pervasive Comput..

[35] Chang Y.F., Ding J.J., Hu H., Yang W.C., Lin K.H., Wu P.H. A real-time detection algorithm for freezing of gait in Parkinson’s disease. Proceedings of the 2014 IEEE International Symposium on Circuits and Systems (ISCAS).

[36] Hollman J.H., McDade E.M., Petersen R.C. (2011). Normative spatiotemporal gait parameters in older adults. Gait Posture.

[37] Alcock L., Galna B., Perkins R., Lord S., Rochester L. (2018). Step length determines minimum toe clearance in older adults and people with Parkinson’s disease. J. Biomech..

[38] Del Din S., Godfrey A., Rochester L. (2016). Validation of an accelerometer to quantify a comprehensive battery of gait characteristics in healthy older adults and Parkinson’s disease: Toward clinical and at home use. IEEE J. Biomed. Health Inform..

[39] Del Din S., Hickey A., Ladha C., Stuart S., Bourke A.K., Esser P., Rochester L., Godfrey A. (2016). Instrumented gait assessment with a single wearable: An introductory tutorial. F1000Research.

[40] Millecamps A., Lowry K.A., Brach J.S., Perera S., Redfern M.S., Sejdić E. (2015). Understanding the effects of pre-processing on extracted signal features from gait accelerometry signals. Comput. Boil. Med..

[41] Kobsar D., Ferber R. (2018). Wearable Sensor Data to Track Subject-Specific Movement Patterns Related to Clinical Outcomes Using a Machine Learning Approach. Sensors.

[42] Saad A. (2016). Detection of Freezing of Gait in Parkinson’s Disease.

[43] Rawassizadeh R., Tomitsch M., Nourizadeh M., Momeni E., Peery A., Ulanova L., Pazzani M. (2015). Energy-efficient integration of continuous context sensing and prediction into smartwatches. Sensors.

[44] Rehman M., Liew C., Wah T., Shuja J., Daghighi B. (2015). Mining personal data using smartphones and wearable devices: A survey. Sensors.

[45] Banaee H., Ahmed M.U., Loutfi A. (2013). Data mining for wearable sensors in health monitoring systems: A review of recent trends and challenges. Sensors.

[46] Howcroft J., Lemaire E.D., Kofman J. (2016). Wearable-sensor-based classification models of faller status in older adults. PLoS ONE.

[47] Castro D., Coral W., Rodriguez C., Cabra J., Colorado J. (2017). Wearable-Based Human Activity Recognition Using an IoT Approach. J. Sens. Actuator Netw..

[48] Sijobert B., Benoussaad M., Denys J., Pissard-Gibollet R., Geny C., Coste C.A. (2015). Implementation and Validation of a Stride Length Estimation Algorithm, Using a Single Basic Inertial Sensor on Healthy Subjects and Patients Suffering from Parkinson’s Disease. Electron. Healthc..

[49] Handojoseno A.A., Shine J.M., Nguyen T.N., Tran Y., Lewis S.J., Nguyen H.T. The detection of Freezing of Gait in Parkinson’s disease patients using EEG signals based on Wavelet decomposition. Proceedings of the 2012 Annual International Conference of the IEEE Engineering in Medicine and Biology Society (EMBC).

